# High-Throughput *MICA/B* Genotyping of Over Two Million Samples: Workflow and Allele Frequencies

**DOI:** 10.3389/fimmu.2020.00314

**Published:** 2020-02-21

**Authors:** Anja Klussmeier, Carolin Massalski, Kathrin Putke, Gesine Schäfer, Jürgen Sauter, Daniel Schefzyk, Jens Pruschke, Jan Hofmann, Daniel Fürst, Raphael Carapito, Seiamak Bahram, Alexander H. Schmidt, Vinzenz Lange

**Affiliations:** ^1^DKMS Life Science Lab, Dresden, Germany; ^2^DKMS, Tübingen, Germany; ^3^Institute of Clinical Transfusion Medicine and Immunogenetics Ulm, German Red Cross Blood Transfusion Service, Baden Wuerttemberg – Hessen, and University Hospital Ulm, Ulm, Germany; ^4^Institute of Transfusion Medicine, University of Ulm, Ulm, Germany; ^5^Laboratoire d'ImmunoRhumatologie Moléculaire, Plateforme GENOMAX, INSERM UMR_S 1109, LabEx TRANSPLANTEX, Université de Strasbourg, Strasbourg, France

**Keywords:** MICA, MICB, hematopoietic stem cell transplantation, allele, genotyping, next generation sequencing, NGS, high-throughput

## Abstract

MICA and MICB are ligands of the NKG2D receptor and thereby influence NK and T cell activity. *MICA/B* gene polymorphisms, expression levels and the amount of soluble MICA/B in the serum have been linked to autoimmune diseases, infections, and cancer. In hematopoietic stem cell transplantation, *MICA* matching between donor and patient has been correlated with reduced acute and chronic graft-vs.-host disease and improved survival. Hence, we developed an extremely cost-efficient high-throughput workflow for genotyping *MICA/B* for newly registered potential stem cell donors. Since mid-2017, we have genotyped over two million samples using NGS amplicon sequencing for *MICA/B* exons 2–5. In donors of German origin, MICA*008 is the most common *MICA* allele with a frequency of 42.3%. It is followed by MICA*002 (11.7%) and MICA*009 (8.8%). The three most common *MICB* alleles are MICB*005 (43.9%), MICB*004 (21.7%), and MICB*002 (18.9%). In general, *MICB* is less diverse than *MICA* and only 6 alleles, instead of 15, account for a cumulative allele frequency of 99.5%. In 0.5% of the samples we observed at least one allele of *MICA* or *MICB* which has so far not been reported to the IPD/IMGT-HLA database. By providing *MICA/B* typed voluntary donors, clinicians become empowered to include *MICA/B* into their donor selection process to further improve unrelated hematopoietic stem cell transplantation.

## Introduction

The *MICA* (MHC class I polypeptide-related sequence A) and *MICB* (MHC class I polypeptide-related sequence B) genes are located between the MHC class I and class III genes inside the human major histocompatibility complex (MHC) (1). Although being highly similar to the classical human leukocyte antigen (*HLA*) genes, they do not present peptides and are not expressed at the surface of human leukocytes but on endothelial cells, fibroblasts, epithelial cells, and tumor cells (2). There they act as ligands for the NKG2D receptor which plays an important role in immune surveillance by activating NK cells and co-stimulating T cell subsets (3, 4). Therefore, the expression of NKG2D ligands is highly regulated and induced by cellular stress (e.g., infection, oxidative stress, transformation).

*MICA* and *MICB* are highly similar and share around 91% of their coding sequence (1). Exon 1 encodes the leader peptide, exons 2, 3, and 4 the three extracellular domains, exon 5 the transmembrane domain and exon 6 the cytoplasmic tail (1, 2, 5). Even though *MICA* and *MICB* do not seem to be as diverse as the conventional *HLA* genes, a large number of distinct alleles have been described: release 3.37.0 of the IPD-IMGT/HLA database contains 109 *MICA* and 47 *MICB* alleles (6). MICA*008 has been reported to be the most common *MICA* allele with frequencies ranging from 25 to 55% depending on the population. Frequencies above 5% were observed for MICA*002, MICA*009, MICA*004, MICA*010, and MICA*007 in Europeans. In Chinese cohorts, the alleles MICA*019, MICA*027, and MICA*045 are also common (7–11). The less diverse *MICB* gene has been predominantly studied in Asian populations. There, the allele MICB*005 is the most common allele with frequencies of over 50%. It is followed by MICB*002 and MICB*004 with frequencies over 10% and MICB*008 and the null allele MICB*009N with frequencies over 5% (10–13).

The most frequent *MICA* allele MICA*008 differs substantially from most other alleles since it lacks the transmembrane domain due to a frameshift in exon 5. Alleles sharing this feature are also referred to as “A5.1” alleles (14). Their products are bound to the cellular membrane by a GPI-anchor and are frequently released into exosomes thereby triggering a systemic downregulation of the NKG2D receptor on effector cells. Other *MICA* and *MICB* alleles do this to a lesser extent using a soluble form caused by a proteolytic shedding mechanism (15, 16). Since high levels of both forms of soluble MICA and MICB (sMICA/B) have been found in various cancers, the release of MIC proteins is thought to be one cause for cancer immune escape. sMICA/B are therefore considered promising targets for immunotherapy (17–20).

Several studies looked into the general impact of *MICA/B* polymorphisms on different diseases. Especially the *MICA*-129Met/Val dimorphism encoded by the SNP rs1051792 has received attention because it separates the different *MICA* alleles into NKG2D-receptor low (Val)- and high (Met)-affinity binding alleles (21). Health risk associations have been shown for several autoimmune diseases, cancer and viral infections (22–27). Furthermore, matching of *MICA*, including the *MICA*-129 dimorphism, between donor and patient has been correlated with improved outcome of unrelated hematopoietic stem cell transplantation and reduced acute and chronic graft-vs.-host disease (28–32). Because *MICA* is in strong linkage disequilibrium with *HLA-B*, over 90% of 10/10 *HLA*-matched donor/patient pairs are also matched for *MICA* (8, 30). In partially matched cases, in particular in *HLA-B* mismatch situations, *MICA* mismatches are more frequent.

To facilitate further studies on *MICA* and/or *MICB* matching in unrelated hematopoietic stem cell transplantation, we included both genes into our high-throughput genotyping workflow for newly registered potential stem cell donors in 2017. This workflow was initially developed for the six classical *HLA* genes *HLA-A, HLA-B, HLA-C, HLA-DRB1, HLA-DQB1*, and *HLA-DPB1* and was then gradually extended to also include *CCR5*, the blood groups *ABO* and *Rh* as well as the several *KIR* genes and *HLA-E* (33–37). Today, this workflow has been applied to genotype over seven million donors, among them more than two million including *MICA* and *MICB*.

## Materials and Methods

### Samples

Volunteers from Germany, Poland, UK, USA, Chile and India provided over two million samples to DKMS for their registration as potential stem cell donors between August 2017 and October 2019. We determined *MICA* and *MICB* allele frequencies based on 1,201,896 samples of donors from DKMS Germany who declared to be of German descent. All subjects gave written informed consent in accordance with the Declaration of Helsinki. The described genotyping is within the scope of the consent forms signed at recruitment and performed as genotyping service.

### DNA Isolation and Quantification

The vast majority of samples were provided as buccal swabs (Copan, Brescia, Italy). Few samples were provided as blood. DNA was isolated using the chemagic™ Blood/Swab Kits (PerkinElmer chemagen Technologie GmbH, Baesweiler, Germany) and quantified by fluorescence as described before (36).

### PCR Amplification

**Figure 1:**
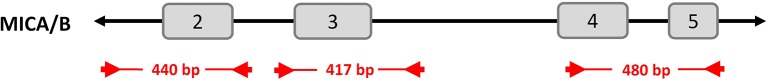
Primer locations and PCR amplification products for exons 2–5 of *MICA/B*. Primers (arrows) bind to both *MICA* and *MICB* and generate three amplicons per gene in one PCR reaction. Product lengths are between 417 and 480 bp. Note that not all bases of exons 4 and 5 are covered.

*MICA* and *MICB* were amplified in one multiplexed PCR reaction targeting exons 2, 3, and 4/5. The resulting amplicons had lengths between 417 and 480 bp (Figure 1). Exons 2 and 3 were amplified as separate amplicons and were completely covered. In contrast, exons 4 and 5 were amplified together as one joined amplicon with primers inside the exons. Therefore, 65 bases at the beginning of exon 4 and 13 bases at the end of exon 5 were not covered. The 8 μl PCR reactions were performed in 384-well plates using FastStart^TM^ Taq DNA Polymerase (Roche, Basel, Switzerland) in its associated buffer system. After amplification, products were pooled with other amplicons of the same sample and subjected to a barcoding/indexing PCR as described previously (33–37).

### Library Preparation and Sequencing

After indexing PCR, 384 barcoded samples were pooled together and purified using SPRIselect beads (BeckmanCoulter, Brea, USA) with a ratio of 0.6:1 beads to DNA and subsequently quantified by qPCR. Equimolar amounts of 10 pools were then combined to a final sequencing library which contained all amplicons from 3,840 donors. The library was denatured and diluted as recommended by Illumina (MiSeq Reagent Kit V2-Reagent Preparation Guide) and loaded at 12.5 pM onto HiSeq flow cells. Paired-end sequencing was performed at 2 × 249 bp using HiSeq Rapid SBS Kits V2 (500 cycles) on HiSeq2500 instruments (Illumina, San Diego, USA) (33–37).

### Genotyping

The neXtype software was extended to support *MICA* and *MICB* genotyping (33, 36). It uses a decision-tree-based algorithm to match the generated *MICA/B* amplicons to known alleles from the official IPD/IMGT-HLA database. Since no known *MICA* amplicon sequence matches a known *MICB* amplicon sequence, reads could be unambiguously assigned to either *MICA* or *MICB*. For more than 95% of the samples neXtype generated correct results with only minor requirements for user interaction. In case of insufficient read coverage, rare or questionable results, a new PCR reaction was initiated from the original DNA. If a low read coverage was limited to exons 4 and 5, trained analysts could decide to generate a result based on exons 2 and 3 only. Genotyping results were finally exported using the GL string format (38).

### Frequency Analysis of *MICA* and *MICB* Alleles

*MICA* and *MICB* genotyping results of 1,201,896 samples of German origin were analyzed based on the first field, which identifies the unique MICA and MICB proteins. Homozygous genotyping results were counted as two alleles. Allele groups which could not be distinguished due to missing sequencing information were reported by a representative allele which was marked with a hash symbol (#) (Table 1). For samples with phasing ambiguities, the probability of each possible result was calculated based on the allele frequencies of unambiguously typed samples. According to these probabilities, counts were added to the different alleles. To verify rare allele calls, all alleles observed <50 times were reconfirmed in at least two samples.

**Table 1:** Overview of ambiguous genotyping results.

**Allele group**	**Alleles**
***MICA***	
MICA*009#	MICA*009, MICA*049
MICA*010#	MICA*010, MICA*065, MICA*069
MICA*027#	MICA*027, MICA*048
***MICB***	
MICB*004#	MICB*004, MICB*028
MICB*005#	MICB*003, MICB*005, MICB*006, MICB*010
MICB*014#	MICB*014, MICB*015

*Alleles which cannot be distinguished from each other by the workflow are combined in an allele group marked with a hash symbol (#)*.

## Results

### High-Throughput *MICA/B* Genotyping

#### Assay Validation and Performance

For assay validation, we exchanged DNA from 95 samples with two labs with established workflows for *MICA* or *MICB* genotyping (*MICA*: Institute of Clinical Transfusion Medicine and Immunogenetics Ulm, Germany; *MICB*: Laboratoire d'ImmunoRhumatologie Moléculaire, Strasbourg, France). For *MICA*, we additionally used the UCLA *MICA* Panel Set (UCLA Immunogenetic Center, USA), which consists of 24 samples with diverse combinations of *MICA* alleles. The results obtained from our newly established workflow were 100% concordant with the reference genotypes for both *MICA* and *MICB* (Supplementary File 1). Subsequently, *MICA/B* genotyping was included into our standard genotyping workflow in August 2017 and applied for all newly registered donors. So far, we have generated *MICA/B* genotyping data for over two million samples, on average more than 20,000 samples per week. Because *MICA/B* amplicons are pooled with the *HLA* amplicons directly after the initial PCR, additional costs for genotyping *MICA/B* are minor and reflect the costs for one 8 μl PCR reaction, sequencing and data analysis. We are targeting an average coverage of 1,000 reads per locus and exon corresponding to a total of 6,000 reads for *MICA/B* with associated costs of about 10 cents per sample for sequencing. This efficient strategy makes it feasible to genotype every newly registered donor for *MICA/B*.

#### Resolution and Ambiguities

Our *MICA/B* genotyping workflow targets and amplifies exons 2 and 3 separately and most of exons 4 and 5 using a combined amplicon (Figure 1). Consequently, exons 1 and 6 and 78 bases of exons 4 and 5 are not sequenced. This amplification strategy promised a good genotyping resolution while being highly cost-efficient. *MICA/B* exons 2, 3, and 5 were considered mandatory because they encode the receptor-interacting domains or define MICA*008-like alleles. Expansion of the exon 5 amplicon made it possible to also include most of exon 4. Exons 1 and 6 encode a leader peptide and the cytoplasmic tail. As these regions do not encode extracellular domains of the proteins and are characterized by a lower diversity they were not included in the genotyping strategy. However, some alleles may only be differentiated by sequence features within one of the not covered regions. For example, SNPs in exon 6 are the only way to distinguish MICA*010 from MICA*069 or MICA*009:01 from MICA*049. MICA*009:02, on the other hand, can be unambiguously genotyped because it differs from MICA*049 and its synonymous allele MICA*009:01 in exon 3 (Table 1) (14). Due to the primer location inside exon 4 our workflow also cannot distinguish between MICA*10 and MICA*065.

For *MICB*, the most common allele MICB*005:02 cannot be distinguished from MICB*003, MICB*006, and MICB*010, while other variants of MICB*005 can be distinguished. Likewise, the pairs MICB*004 and MICA*028 or MICB*014 and MICA*015 cannot be resolved (Table 1).

In addition to the ambiguities caused by missing sequence information, we encounter phasing ambiguities. They occur because the sequences of short amplicons cannot be phased if the targeted regions are not overlapping. As a consequence, some observed sequence combinations can be explained by more than one allele pair. In our workflow, phasing ambiguities occur in 3% of *MICA* and 24% of *MICB* samples. In over 99.9% of those cases, however, one possibility can statistically be ruled out since the combination of two rare alleles would be highly unlikely if the other option includes two common alleles. This is in contrast to *HLA* genotyping where some important phasing ambiguities cannot be solved statistically. For example, the most common *MICB* phasing ambiguity result is either the combination MICB*002 and MICB*005# or the combination MICB*018 and MICB*019 [GL-String notation: MICB*002+MICB*005#|MICB*018+MICB*019 (38)]. Based on the allele frequencies determined in this study, the likelihood of the allele combination MICB*002+MICB*005# is 0.039. In contrast, the likelihood of MICB*018+MICB*019 is only 1.9 × 10^−8^. Hence, MICB*018+MICB*019 would be expected to occur only once in 2.08 million samples with the given phasing result. In our dataset of 1,201,896 samples, 182,383 samples have the result MICB*002+MICB*005#|MICB*018+MICB*019. Now, by claiming that MICB*002+MICB*005# is always the correct result, we are making only one wrong call in 13.7 million genotyped samples. Therefore, we have disregarded the highly unlikely combinations of rare alleles in our allele frequency calculations. This is not expected to introduce a relevant error. In contrast, disregarding all samples with phasing results altogether would substantially distort the results since the phasing events predominantly involve certain alleles.

#### Novel Alleles

We encounter novel *MICA* or *MICB* alleles in 0.5% of the samples, resulting in the observation of ~100 novel alleles per week (recurrences included). They are automatically flagged by the genotyping software and trigger a new PCR reaction from the original sample for verification. In general, the novel alleles fall into two categories: Novel sequences or novel combinations of previously reported exonic sequences. The task to characterize them in full length and submit the sequences to IPD/IMGT-HLA is currently in progress.

### *MICA* Allele Frequencies

**Figure 2:**
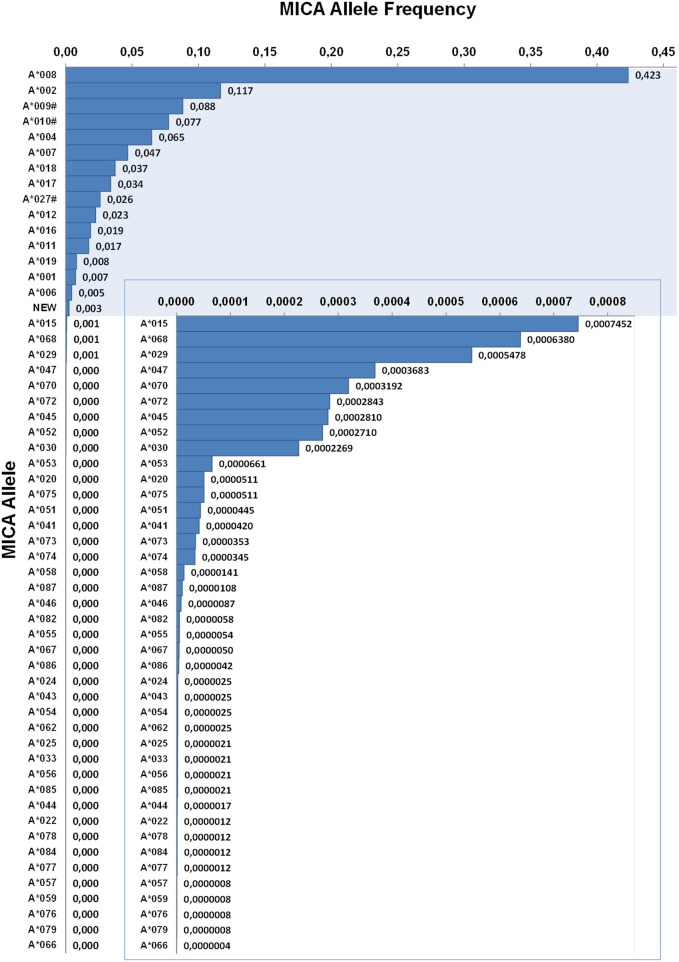
Allele frequencies of *MICA*. First-field-resolution allele frequencies are based on 1,201,896 samples from donors of German descent. Alleles contributing to a cumulative allele frequency of 99.5% are shown against a colored background and allele frequencies below 0.003 are additionally plotted in an inlay. If ambiguities exist, allele groups are used (#) and the ambiguity is described in Table 1.

*MICA* allele frequencies were calculated on 1,201,896 samples of German descent (Figure 2). These samples represent more than 50% of our genotyped samples and were therefore the largest ethnically defined population available. With a frequency of 42.3%, the allele MICA*008 is the most frequent *MICA* allele in Germany. It is followed by the alleles MICA*002 (11.7%), MICA*009# (8.8%), MICA*010# (7.7%), and MICA*004 (6.5%). The 15 most common alleles account for a cumulative allele frequency of 99.5%. The other 41 alleles observed in the German dataset account for the remaining 0.5%. We further identified six *MICA* alleles (MICA*035, MICA*037, MICA*038, MICA*040, MICA*060, and MICA*064N) with very low frequencies in samples not of German origin. Despite the huge sample size, we have never observed the remaining 18 alleles contained in the IPD-IMGT/HLA database (release 3.37.0) (Table 2).

**Table 2:** *MICA/B* alleles described in IPD/IMGT-HLA release 3.37.0, but never observed in our cohort of over two million samples.

*MICA*	MICA*005, MICA*013, MICA*014, MICA*023, MICA*026, MICA*028, MICA*031, MICA*032, MICA*034, MICA*036, MICA*039, MICA*042, MICA*050, MICA*061, MICA*063N, MICA*065, MICA*081, MICA*083
*MICB*	MICB*001, MICB*011, MICB*016, MICB*022, MICB*030, MICB*032

### *MICB* Allele Frequencies

**Figure 3:**
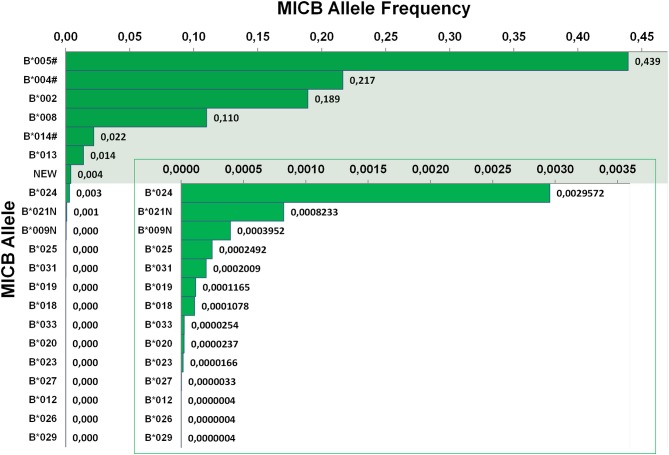
Allele frequencies of *MICB*. First-field-resolution allele frequencies are based on 1,201,896 samples from donors of German descent. Alleles contributing to a cumulative allele frequency of 99.5% are shown against a colored background and allele frequencies below 0.004 are additionally plotted in an inlay. If ambiguities exist, allele groups are used (#) and the ambiguity is described in Table 1.

*MICB* allele frequencies were calculated based on the same sample cohort used for *MICA* (Figure 3). With a frequency of 43.9%, MICB*005# is by far the most frequent allele in Germany. However, since our workflow cannot distinguish all MICB*005 variants from MICB*003, MICB*006, and MICB*010, the true frequency of MICB*005 might be lower (Table 1). In our samples, MICB*005# is followed by MICB*004#, MICB*002, and MICB*008, having frequencies of 21.7 18.9, and 11.0%, respectively. Together with MICB*014# (2.2%) and MICB*013 (1.4%) they account for a cumulative allele frequency of 99.5%. 14 other alleles have been detected in the German cohort. MICB*007 has only been identified in a few samples of non-German origin. We have never observed the six remaining alleles described in the IPD-IMGT/HLA database (release 3.37.0) (Table 2).

## Discussion

The regulation of NK/T cell activation is an elaborate interplay between several receptors and their associated ligands. To further add another layer of complexity, receptors like KIR or ligands like MICA/B exist in a variety of distinct alleles with varying effects on NK/T cell activity (6, 39). A comprehensive sequencing study of the MHC complex indicated that the sequence of *MICA* is more diverse than the sequence of *HLA-DQB1* or *HLA-DPB1*, but the number of named *MICA* alleles is much lower (6, 10). And even though MICA/B do not present antigenic peptides like the classical *HLA* class I genes, matching of *MICA/B* between patient and donor has been reported to improve outcome and reduce acute and chronic graft-vs.-host disease in hematopoietic stem cell transplantation, especially in partially matched scenarios (30, 31, 40). Translation of these findings into clinical practice is, amongst others, hampered by the lack of *MICA/B* genotyping data. Hence, we present a workflow to genotype both *MICA* and *MICB* with a mean throughput of over 20,000 samples per week. To date, we have processed more than two million donor samples.

Based on 1.2 million samples of German origin we identified MICA*008 as the most common *MICA* allele (42.3%), followed by MICA*002 (11.7%) and MICA*009# (8.8%). This is concordant to previous studies which present allele frequencies between 43 and 55% for MICA*008, 8–14% for MICA*002 and 4–8% for MICA*009 in European/American populations (7–9). Although MICA*008 is also the most common allele in China, with a frequency of about 25% it is far less abundant than in European/American populations (10, 11, 41). Since MICA*008 and other rare alleles bearing the A5.1 microsatellite marker are more prone to produce sMICA than other alleles, they are more effective in inactivating NKG2D and NK/T cell activity (15). Therefore, these alleles might contribute to the disease prevalence in different populations. Indeed, A5.1-carriers have been associated with an increased risk for several types of cancer and higher levels of sMICA seem to have a negative prognostic value for tumor patient survival (18, 27, 42–44). To reactivate a patient's NK cells, the reduction of soluble NKG2D ligands is a promising approach. Current strategies comprise the inhibition of enzymes responsible for shedding as well as blocking the cleavage sites with therapeutic antibodies. Most likely, the efficacy of some of these new drugs will be limited to certain *MICA/B* alleles which increases the need for reliable genotyping (18, 45).

*MICB* is less diverse than *MICA*. The most common allele MICB*005# was detected at 43.9% allele frequency in the German population. However, given the incomplete sequence coverage, our workflow cannot distinguish MICB*003, MICB*005, MICB*006, and MICB*010. Studies on Asian cohorts report allele frequencies of at least 55% for MICB*005, 3% for MICB*003 and no observations of MICB*006 or MICB*010 (10, 11, 13). Limited full gene analysis of 51 samples with MICB*005# pre-typing results indicated a similar distribution in our dataset (data not shown).

The MICB*003/005:02 ambiguity with its distinguishing bases at the beginning of exon 4 and in exon 6 is one case in which our workflow cannot differentiate between two presumably common alleles. However, an amplicon of at least 530 bp would be necessary to include the SNP at the beginning of exon 4 and to not lose sequencing information for the microsatellite region in *MICA* exon 5. Since this exceeds Illumina's 2 × 250 bp read length, bases at the end of exon 4 would not be sequenced, thereby creating other ambiguities. Consequently, to clearly distinguish between MICB*005:02 and MICB*003 a separate fourth PCR amplicon would be required. But given the lack of clinical data for the relevance of regions outside exons 2, 3, and 5, one might wonder if a higher resolution for *MICA/B* genotyping is necessary. In *HLA* genotyping transplantation compatible allele groups have been defined (G or P Codes) combining all alleles harboring the same sequence across the antigen recognition domain (2, 46, 47). For *MICA/B*, there is no similar system yet. Consequently, we do not think that it is proportionate to increase the sequencing costs for all samples without further evidence of the clinical importance of remaining ambiguities. For individual samples, genotyping results with three-field resolution can be generated using long-read sequencing technologies (48). Moreover, our amplicon strategy does not include the 5′ and 3′ UTRs of *MICA/B* which contain additional polymorphic positions (49, 50). Some of them influence (s)MICA/B expression which varies between different alleles (18, 51–53). However, to the best of our knowledge, there are no studies, which address the effects of donor *MICA/B* variations outside the exons in hematopoietic stem cell transplantation.

Although we genotyped over two million samples, we have not encountered some of the *MICA/B* alleles described in the IPD/IMGT-HLA database (Table 2). This may be due to several reasons. First of all, the majority of our samples are of European origin. Therefore, we might lack rare alleles occurring predominantly in other ethnicities. One example is MICB*032 which was originally isolated from an Uyghur individual (54). In other cases, initial submissions to IPD/IMGT-HLA could be erroneous. This might especially be true for the alleles that have never been independently confirmed. For example, all heterozygous positions defining MICA*005 or MICA*013 also occur in one of the two most common alleles MICA*008 and MICA*002. If those positions were not correctly phased during Sanger sequence analysis, MICA*005 and MICA*013 could have been erroneously reported. However, the sequencing of cloned PCR fragments should have prevented such errors (1, 55). Other not observed alleles, like MICA*081, MICB*011, MICB*016, or MICB*022, differ from more common alleles in only one position (56, 57). While this may reflect sequencing errors, it is more likely that the more recent submissions represent very low frequency observations as we discover on a daily basis. However, for the individual allele this may only be resolved by resequencing the original DNA which is often no longer available.

In conclusion, our workflow demonstrates that upfront *MICA/B* genotyping for potential stem cell donors can be performed with only minor increases in expenses and workload. So far, *MICA/B* informed donor selection has not yet found widespread application in clinical practice. Clearly, additional confirmatory studies would be worthwhile. However, the availability of genotyping information remains a major hurdle for the translation of new markers into clinical practice. With the *MICA/B* genotyping of millions of donors we provide that data to facilitate *MICA/B* informed donor selection.

## Data Availability Statement

The raw data supporting the conclusions of this article will be made available by the authors, without undue reservation, to any qualified researcher.

## Ethics Statement

Ethical review and approval was not required for the study on human participants in accordance with the local legislation and institutional requirements. The patients/participants provided their written informed consent to participate in this study.

## Author Contributions

KP developed and tested the primer set. JS, DS, JP, and JH developed and implemented the genotyping algorithm. DF, RC, and SB performed genotyping for reference samples. AK, CM, GS, and JS analyzed frequency data. AK prepared figures and tables. AK and VL wrote the manuscript. AK, AS, and VL conceived and supervised the work. All authors contributed to manuscript revision, read, and approved the submitted version.

### Conflict of Interest

AK, CM, GS, KP, AS, and VL are members of the DKMS Life Science Lab which offers commercial genotyping services. The remaining authors declare that the research was conducted in the absence of any commercial or financial relationships that could be construed as a potential conflict of interest.
